# Knowledge, Awareness, and Perception of Advanced Medical Directives Among Patients and Their Relatives in the Critical Care Unit: A Pilot Study

**DOI:** 10.7759/cureus.80538

**Published:** 2025-03-13

**Authors:** Ajay Kumar, Jyoti Barwa, Rattan Singh, Sahil Thakral, Apurba Patra

**Affiliations:** 1 Forensic Medicine and Toxicology, All India Institute of Medical Sciences, Bathinda, Bathinda, IND; 2 Anatomy, All India Institute of Medical Sciences, Bathinda, Bathinda, IND

**Keywords:** advance medical directives, death, do-not-resuscitate, healthcare, legislation as topic, palliative care

## Abstract

Background: Advance medical directives (AMDs) are legal documents that allow individuals to specify their medical care preferences in case they become incapacitated due to severe illness or injury. Understanding public awareness and attitudes toward AMDs is crucial for improving their implementation.

Materials and methods: A cross-sectional study was conducted in the critical care unit of a tertiary care hospital, involving 50 patients and their relatives over the age of 18 after taking informed written consent. A specially designed questionnaire was used to assess their awareness, understanding, and attitudes toward AMDs.

Results: The study revealed significant gaps in awareness and adoption of AMDs. Notably, none of the female participants had prepared the AMDs, and all individuals who had prepared one were over the age of 60. Additionally, none of the Hindu participants had AMDs, while three (8.1%) Sikh participants had prepared one. Awareness of legal formalities, such as the need for notarization or witnessing, was low. Moreover, only a minority of participants were aware that healthcare institutions could decline to comply with an AMD.

Conclusions: While some awareness of AMDs exists, the majority of the population remains unfamiliar with their guidelines and procedures. Targeted educational initiatives are essential to bridge this knowledge gap and promote informed decision-making regarding end-of-life care.

## Introduction

The healthcare system in India is rapidly evolving with advancements in medicine, surgery, and artificial intelligence, along with the enactment of new laws. However, public access to these developments remains limited [1]. Currently, healthcare expenses in India are primarily paid out of pocket, as in many other low-income nations [2]. The average Indian household spends between 5% and 7% of its annual income on healthcare, and for 15-20% of households, healthcare costs exceed 10% annually, a level considered catastrophic. The cost of end-of-life care is rising and is often unaffordable [3]. The COVID-19 pandemic affected both developed and developing nations alike, underscoring the importance of early healthcare planning and the timely preparation of a living will. Over the past few decades, as awareness of life’s unpredictability has grown, advance medical directives (AMDs) have gained global recognition. When these directives were introduced in the United States in the late 1960s, they were swiftly incorporated into American law. In 1976, California became the first state to enact legislation in this area [4]. Following advancements in North America, healthcare directives have gradually been adopted in other countries, particularly those influenced by North American culture, such as Canada, Australia, New Zealand, Japan, and Singapore [5-9]. However, China faced significant challenges implementing AMDs [10]. In contrast, Europe has slowly embraced this trend [11].

In India, the Honourable Supreme Court’s landmark ruling in the 1978 case of Maneka Gandhi vs. Union of India established the legal foundation for AMDs by interpreting Article 21 of the Constitution of India to encompass dignity and a meaningful, fulfilling life [12]. However, in 2018, the Supreme Court set forth principles governing the execution of AMDs, or "living wills," providing guidelines and safeguards [13,14]. Due to the intricate process of registering and executing AMDs, the Indian Society of Critical Care Medicine approached the Supreme Court in 2018 for clarification in the Common Cause vs. Union of India and Another case [15].

As a result, the Supreme Court issued the "2023 Order," which introduced the following changes concerning AMDs [16]: (1) attestation of AMDs before a notary or gazetted officer instead of a judicial magistrate, (2) greater flexibility in appointing guardians or close relatives, (3) relaxation of qualifications for appointing primary and secondary boards, and (4) prescribed time limits for swift action and maintenance of digital health records along with simplification of procedural and implementation requirements.

In the Indian context, AMDs can only be executed by a mentally competent adult who fully understands their implications [15,16]. Furthermore, the AMDs must be voluntary, free from coercion or undue influence, no matter how subtle. Honoring a patient’s autonomy entails ethical responsibilities and strengthens their independence [17]. In doing so, physicians acknowledge and respect an individual’s capacity to make informed decisions aligned with their values and beliefs. This study aimed to assess individuals' comprehension, awareness, and attitudes toward AMDs using a specially designed questionnaire. Since the concept of AMDs is relatively new in India, awareness and understanding were presumed to remain low within the community.

## Materials and methods

A cross-sectional pilot study was conducted in a tertiary care hospital's critical care unit after receiving approval from the Institutional Ethics Committee of the All India Institute of Medical Sciences, Bathinda (approval number: IEC/AIIMS/BTI/355, approval date: April 26, 2023). As this was a pilot study, purposive sampling with only 50 samples and continuous admissions in the critical care unit of the tertiary care hospital (surgical intensive care unit, medical intensive care unit, and coronary care unit) was taken.

Inclusion and exclusion criteria

All adult patients (or their relatives), male or female, greater than 18 years of age, who were admitted to the critical care unit of a tertiary care hospital, giving written informed consent to participate in the study, were included. Either the patient, if deemed fit to respond, or one of their close relatives was included as a study participant. Mentally ill patients and their relatives, patients under 18 years old, or patients who refused consent were excluded from the study.

Study questionnaire

Data was collected using a specially designed questionnaire tailored to the study objectives. The questionnaire was completed after obtaining written informed consent from the patients or their relatives. All questions were single-option questions. For participants who had difficulty understanding the questions, the principal investigator or co-principal investigators provided explanations before recording their responses. The questionnaire included socio-demographic information and assessed participants' knowledge, attitudes, and perceptions of AMDs.

Statistical analysis

The data were collected and entered into Microsoft Excel 2010 (Microsoft Corporation, Redmond, WA, USA). Various statistical analyses were performed using R software version 4.0.2 (Foundation for Statistical Computing, Vienna, Austria, https://www.R-project.org/). The one-sample Kolmogorov-Smirnov test was used to determine if the data sets differed from a normal distribution. Normally distributed data were analyzed using parametric tests, while non-normally distributed data were analyzed using non-parametric tests. The awareness score about AMDs was calculated by summing up the score of individual questions related to awareness of AMD. One mark was assigned for each correct answer. A total of 15 questions were considered for deriving the awareness scale. Furthermore, we have categorized the awareness score into two categories based on the median (<11 and ≥11).

Descriptive statistics were calculated for qualitative and categorical variables. Logistic regression was applied to examine relationships between dependent and independent variables, with results obtained by exponentiating regression coefficients. A p-value of <0.05 was considered statistically significant, whereas a p-value >0.05 was considered statistically insignificant.

## Results

A demographic breakdown of the study participants is shown in Table 1. Among the participants, 39 (78%) were from rural areas, and 37 (74%) had a family income of less than Rs. 30,000. In terms of occupation, 17 (34%) were farmers, 14 (28%) were unemployed, 14 (28%) worked in the private sector, and eight (16%) were employed in the government sector. Regarding admission in the hospital's critical care unit, 18 (36%) were in the surgical intensive care unit, 20 (40%) in the medical intensive care unit, and 12 (24%) in the coronary care unit. Education-wise, 17 (34%) were illiterate, 22 (44%) had completed upper primary schooling, nine (18%) had passed high school, and two (4%) were graduates.

**Table 1:** Socio-demographic variables and association with AMDs among patients admitted in the critical care unit A p-value of <0.05 was considered statistically significant, whereas a p-value >0.05 was considered statistically insignificant AMDs: advance medical directives, Rs: Indian Rupees

Variables name	Categories	Awareness score of AMDs (%)		Total N=50 (%)	p-value
		<11 (n=27)	≥11 (n=23)		
Age (years)	18-40	1 (3.7%)	4 (17.4%)	5 (10%)	0.27
	41-60	23 (85.2%)	17 (73.9%)	40 (80%)	
	>60	3 (11.1%)	2 (8.7%)	5 (10%)	
Gender	Male	24 (88.9%)	18 (78.3%)	42 (84%)	0.03
	Female	3 (11.1%)	5 (21.7%)	8 (16%)	
Literate	Illiterate	9 (33.3%)	8 (34.8%)	17 (34%)	0.13
	Upper primary	15 (55.6%)	7 (30.4%)	22 (44%)	
	High school	3 (11.1%)	6 (26.1%)	9 (18%)	
	Graduate	0 (0%)	2 (8.7%)	2 (4%)	
Profession	Unemployed	6 (22.2%)	8 (34.8%)	14 (28%)	0.67
	Private sector	6 (22.2%)	5 (21.7%)	11 (22%)	
	Government sector	4 (14.8%)	4 (17.4%)	8 (16%)	
	Farmers	11 (40.7%)	6 (26.1%)	17 (34%)	
Monthly income (Rs)	Nil	0 (0%)	2 (8.7%)	2 (4%)	0.18
	<10000	8 (29.6%)	12 (52.2%)	20 (40%)	
	10000-30000	12 (44.4%)	5 (21.7%)	17 (34%)	
	30000-50000	5 (18.5%)	3 (13%)	8 (16%)	
	50000-70000	2 (7.4%)	1 (4.3%)	3 (6%)	
Currently residence	Rural	17 (63%)	22 (95.7%)	39 (78%)	0.01
	Urban	10 (37%)	1 (4.3%)	11 (22%)	
Marital status	Unmarried	4 (14.8%)	4 (17.4%)	8 (16%)	0.14
	Married	23 (85.2%)	16 (69.6%)	39 (78%)	
	Divorced	0 (0%)	3 (13%)	3 (6%)	

The significance level of socio-demographic variables with AMDs is shown in Table 2.

**Table 2:** Significance level of socio-demographic variables A p-value of <0.05 was considered statistically significant, whereas a p-value >0.05 was considered statistically insignificant. NA: not applicable, Rs: Indian Rupees

Variables name	Categories	Odds ratio (upper-lower bound)	p-value
				Age groups	18-40 yrs	6.00 (0.35-101.56)	0.21
41-60 yrs	1.11 (0.17-7.40)	0.91		
>60 yrs	NA	NA		
Gender	Male	0.45 (0.10-2.13)	0.32
	Female	NA	NA
Profession	Unemployed	2.44 (0.57-10.45)	0.23
	Private sector	1.53 (0.33-7.19)	0.59
	Government sector	1.83 (0.33-10.10)	0.49
	Farmers	NA	NA
Monthly income (Rs)	<10000	3.00 (0.23-38.88)	0.4
	10000-30000	0.83 (0.06-11.41)	0.89
	30000-50000	1.20 (0.07-19.63)	0.9
	50000-70000	NA	NA
Residence	Rural	12.94 (1.50-111.19)	0.02
	Urban	NA	NA

In our study, we observed that the majority of participants were male, 42 (84%), with only eight (16%) female participants; only three (7.1%) of the males and none of the females had prepared AMDs. All individuals who had prepared this document belonged to the age group above 60 years. Among those who had not yet prepared AMDs, nine (21.4%) of the males and one (12.5%) of the females intended to prepare AMDs in the future (Table 3).

**Table 3:** Distribution of participants with respect to sex, age, and religion for assessing knowledge, attitude, and perception of AMDs AMDs: advance medical directives, M: male, F: female

Questions	Sex		Age in years			Religion	
	M n=42 (%)	F n=8 (%)	18-40 n=5 (%)	41-60 n=40 (%)	>60 n=5 (%)	Sikh n=37 (%)	HIndu n=13 (%)
1. Do you have AMDs framed for yourself							
Yes	3 (7.1%)	0 (0%)	0 (0%)	0 (0%)	3 (60.0%)	3 (8.1%)	0 (0%)
No	30 (71.4%)	7 (87.5%)	4 (80%)	31 (77.5%)	2 (40.0%)	25 (67.6%)	12 (9.3%)
Will prepare in future	9 (21.4%)	1 (12.5%)	1 (10%)	9 (22.5%)	0 (0%)	9 (24.3%)	1 (7.7%)
2. Can I cancel/modify my AMDs after it is framed?							
Yes	17 (40.5%)	0 (0%)	2 (40.0%)	13 (32.5%)	2 (40.0%)	12 (32.4%)	5 (38.5%)
No	19 (45.2%)	0 (0%)	1 (20.0%)	17 (42.5.0%)	1 (20.0%)	16 (43.2%)	3 (23.1%)
I don’t know	6 (14.3%)	8 (100%)	2 (40.0%)	10 (25.0%)	2 (40.0%)	9 (24.3%)	5 (38.5%)
3. Where should I keep my AMDs							
Home	19 (45.2%)	4 (50.0%)	2 (40.0%)	16 (40.0%)	5 (100%)	18 (48.6%)	5 (38.5%)
Lawyer	0 (0%)	1 (12.5%)	0 (0%)	1 (2.5%)	0 (0%)	1 (2.7%)	0 (0%)
Relative	3 (7.1%)	0 (0%)	1 (20.0%)	2 (5.0%)	0 (0%)	3 (8.1%)	0 (0%)
Doctor	8 (19.1%)	0 (0%)	0 (0%)	8 (20.0%)	0 (0%)	6 (16.2%)	2 (15.4%)
I don’t know	12 (28.6%)	3 (37.5%)	2 (40.0%)	13 (32.5%)	0 (0%)	9 (24.3%)	6 (46.1%)
4. Have you talked/discussed with your family members or relatives about AMDs							
Yes	17 (40.5%)	0 (0%)	1 (20.0%)	12 (30.0%)	3 (60.0%)	17 (45.9%)	0 (0%)
No	25 (59.5%)	8 (100%)	4 (80.0%)	27 (70.0%)	2 (40.0%)	20 (54.1%)	13 (100%)
5. Have you talked/discussed with your family physician about AMDs							
Yes	9 (21.4%)	0 (0%)	1 (20.0%)	8 (20.0%)	0 (0%)	9 (24.3%)	0 (0%)
No	25 (59.5%)	8 (100%)	3 (60.0%)	25 (62.5%)	5 (100%)	20 (54.1%)	13 (100%)
I don’t know	8 (19.1%)	0 (0%)	1 (20.0%)	7 (17.5%)	0 (0%)	8 (21.6%)	0 (0%)
6. Do I need to have my AMDs witnessed or notarized?							
Yes	16 (38.1%)	3 (37.5%)	3 (60.0%)	14 (35.0%)	2 (40.0%)	14 (37.9%)	5 (38.5%)
No	17 (40.5%)	0 (0%)	1 (20.0%)	16 (40.0%)	0 (00%)	17 (45.9%)	0 (0%)
I don’t know	9 (21.4%)	5 (62.5%)	1 (20.0%)	10 (25.0%)	3 (60.0%)	6 (16.2%)	8 (61.5%)
7. Can a healthcare institution refuse to follow my AMDs?							
Yes	2 (4.8%)	0 (0%)	0 (0%)	2 (5.0%)	0 (0%)	2 (5.4%)	0 (0%)
No	25 (59.5%)	5 (62.5%)	2 (40.0%)	28 (70.0%)	0 (0%)	20 (54.1%)	10 (76.9%)
I don’t know	15 (35.7%)	3 (37.5%)	3 (60.0%)	10 (25.0%)	5 (100%)	15 (40.5%)	3 (23.1%)
8. Do you know that the Supreme Court of India has given guidelines for AMDs							
Yes	16 (38.1%)	0 (0%)	1 (20.0%)	13 (32.5%)	2 (40.0%)	14 (37.9%)	2 (15.4%)
No	11 (26.2%)	3 (37.5%)	2 (40.0%)	9 (22.5%)	3 (60.0%)	11 (29.7%)	3 (23.1%)
I don’t know	15 (35.7%)	5 (62.5%)	2 (40.0%)	18 (45.0%)	0 (0%)	12 (32.4%)	8 (61.5%)
9. At any time in your life, your health may deteriorate, and you may not be able to make decisions about your medical care. Have you ever considered the possibility that such a situation could arise?							
Yes	25 (59.5%)	0 (0%)	1 (20.0%)	19 (47.5%)	5 (100%)	17 (45.9%)	8 (61.5%)
No	17 (40.5%)	8 (100%)	4 (80.0%)	21 (52.5%)	0 (0%)	20 (54.1%)	5 (38.5%)
10. Will you consider "Do not resuscitate" (DNR) as one of the AMDs							
Yes	23 (54.8%)	3 (37.5%)	2 (40.0%)	21 (52.5%)	3 (60.0%)	20 (54.1%)	6 (46.1%)
No	10 (23.8%)	0 (0%)	1 (20.0%)	7 (17.5%)	2 (40.0%)	8 (21.6%)	2 (15.4%)
I don’t know	9 (21.4%)	5 (62.5%)	2 (40.0%)	12 (30.0%)	0 (0%)	9 (24.3%)	5 (38.5%)
11. Whom would you like to choose as a healthcare proxy?							
Parents	14 (33.4%)	0 (0%)	2 (40.0%)	11 (27.5%)	1 (20.0%)	14 (37.9%)	0 (0%)
Son/daughter	13 (31%)	3 (37.5%)	1 (20.0%)	13 (32.5%)	2 (40.0%)	9 (24.3%)	7 (53.8%)
Close relative	9 (21.4%)	0 (0%)	1 (20.0%)	7 (17.5%)	1 (20.0%)	8 (21.6%)	1 (7.7%)
Doctor	3 (7.1%)	2 (25%)	0 (0%)	4 (10.0%)	1 (20.0%)	3 (8.1%)	2 (15.4%)
No healthcare proxy	3 (7.1%)	3 (37.5%)	1 (20.0%)	5 (12.5%)	0 (0%)	3 (8.1%)	3 (23.1%)
12. If you had irreversibly lost your mental functions and were able to live only with artificial nutrition and breathing apparatus, would you like to continue life-sustaining treatment?							
Yes	25 (59.5%)	5 (62.5%)	3 (60.0%)	25 (62.5%)	2 (40.0%)	20 (54.1%)	10 (76.9%)
No, let nature take its course	17 (40.5%)	3 (37.5%)	2 (40.0%)	15 (37.5%)	3 (60.0%)	17 (45.9%)	3 (23.1%)
13. Do you want your decision or your family’s decision about AMDs to be considered concerning organ donation?							
Yes	27 (64.3%)	2 (25%)	2 (40.0%)	22 (55.0%)	5 (100%)	19 (51.4%)	10 (76.9%)
No	4 (9.5%)	3 (37.5%)	1 (20.0%)	6 (15.0%)	0 (0%)	7 (18.9%)	0 (0%)
I don’t know	11 (26.2%)	3 (37.5%)	2 (20.0%)	12 (30.0%)	0 (0%)	11 (29.7%)	3 (23.1%)
14. Reason for not getting AMDs framed by you/your patient							
Ignorance	12 (28.6%)	0 (0%)	1 (20.0%)	10 (25.0%)	1 (20.0%)	9 (24.3%)	3 (23.1%)
Costly	3 (7.1%)	0 (0%)	0 (0%)	0 (0%)	3 (60.0%)	3 (8.1%)	0 (0%)
Involve legal proceeding	11 (26.2%)	1 (12.5%)	1 (20.0%)	11 (27.5%)	0 (0%)	8 (21.6%)	4 (30.8%)
Never thought of framing AMDs	16 (38.1%)	7 (87.5%)	3 (60.0%)	19 (47.5%)	1 (20.0%)	17 (45.9%)	6 (46.1%)
15. Will you help or motivate others, including family members, in framing their AMDs							
Yes	18 (42.9%)	2 (25.0%)	1 (20.0%)	14 (45.0%)	5 (100%)	13 (35.2%)	7 (53.8%)
No	15 (35.7%)	3 (37.5%)	2 (40.0%)	16 (40.0%)	0 (0%)	15 (40.5%)	3 (23.1%)
I don’t know	9 (21.4%)	3 (37.5%)	2 (40.0%)	10 (15.0%)	0 (0%)	9 (24.3%)	3 (23.1%)

Considering the religious beliefs of the participants, none (0%) of the Hindu participants had prepared an AMD, whereas three (8.1%) of the Sikh participants had already done so (Table 3). Regarding the cancellation of AMDs, although 17 (40.5%) of males were aware of the possibility, all female participants were completely unaware. Among religious groups, 12 (32.4%) Sikhs and five (38.5%) Hindus were mindful of AMD cancellation. When asked where an AMD should be kept, most male participants (19, 45.2%) were unaware. However, most of those above 60 years (5, 100%) and Sikhs (18, 48.6%) considered keeping their AMDs home. Regarding discussions with family members about AMD preparation, 25 (59.5%) male participants, eight (100%) female participants, and 13 (100%) Hindus had not discussed it at home.

Among professional groups, only three (17.6%) farmers had prepared an AMD, while those in other professions had not. On comparing the educational background, three (17.6%) illiterate participants had prepared their AMDs, whereas none of the literate persons had prepared it (Table 4). Most government employees (5, 62.5%) within professional groups acknowledged the possibility of cancellation. In contrast, among education groups, two (100%) graduates agreed that an AMD could be modified or revoked after its creation. Six (75%) government employees and nearly half of literates (10, 45.5%) had discussed AMDs with their family members or relatives. However, none of the female participants, those over 60 years old, Hindus, or farmers had discussed AMDs with their family physician. Approximately five (62.5%) government and six (54.5%) private sector employees knew that AMDs must be notarized or witnessed.

**Table 4:** Distribution of participants with respect to professional and educational status for assessing knowledge, attitude, and perception of AMDs AMDs: advance medical directives

Questions	Professional				Educational status			
	Farmer n=17 (%)	Government sector n=8 (%)	Private sector n=11 (%)	Unemployed n=14 (%)	Illiterate n=17 (%)	Literate n=22 (%)	High school n=9 (%)	Graduate n=2 (%)
1. Do you have AMDs framed for yourself								
Yes	3 (17.6%)	0 (0%)	0 (0%)	0 (0%)	3 (17.6%)	0 (0%)	0 (0%)	0 (0%)
No	10 (58.8%)	5 (62.5%)	11 (100%)	11 (78.6%)	11 (64.7%)	19 (84.2%)	7 (78.6%)	0 (0%)
Will prepare in the future	4 (23.6%)	3 (37.5%)	0 (0%)	3 (21.4%)	3 (11.7%)	3 (15.8%)	2 (21.4%)	2 (100%)
2. Can I cancel/modify my AMDs after it is framed?								
Yes	5 (29.2%)	5 (62.5%)	3 (27.4%)	4 (28.5%)	5 (29.4%)	6 (27.2%)	4 (44.4%)	2 (100%)
No	9 (53.2%)	0 (0%)	4 (36.3%)	6 (43.0%)	9 (53.0%)	9 (41.0%)	1 (11.2%)	0 (0%)
I don’t know	3 (17.6%)	3 (37.5%)	4 (36.3%)	4 (28.5%)	3 (17.6%)	7 (31.8%)	4 (44.4%)	0 (0%)
3. Where should I keep my AMDs?								
Home	13 (76.6%)	8 (100%)	0 (0%)	2 (14.3%)	8 (47.0%)	10 (45.5%)	4 (44.4%)	1 (50.0%)
Lawyer	0 (0%)	0 (0%)	0 (0%)	1 (7.1%)	1 (5.9%)	0 (0%)	0 (0%)	0 (0%)
Relative	0 (0%)	0 (0%)	2 (18.1%)	1 (7.1%)	1 (5.9%)	2 (9.1%)	0 (0%)	0 (0%)
Doctor	0 (0%)	0 (0%)	6 (54.5%)	2 (14.3%)	2 (11.8%)	5 (22.7%)	0 (0%)	1 (50.0%)
I don’t know	4 (23.4%)	0 (0%)	3 (27.4%)	8 (57.2%)	5 (29.4%)	5 (22.7%)	5 (55.6%)	0 (0%)
4. Have you talked/discussed with your family members or relatives about your AMDs								
Yes	6 (35.3%)	6 (75%)	3 (27.4%)	2 (14.3%)	5 (29.4%)	10 (45.5%)	1 (11.2%)	1 (50.0%)
No	11 (67.7%)	2 (25%)	8 (72.6%)	12 (85.7%)	12 (70.6%)	12 (54.5%)	8 (88.8%)	1 (50.0%)
5. Have you talked/discussed with your family physician about your AMDs								
Yes	3 (17.6%)	3 (75.0%)	1 (9.2%)	2 (14.3%)	0 (0%)	7 (31.8%)	1 (11.2%)	1 (50.0%)
No	12 (70.6%)	5 (25.0%)	4 (36.3%)	12 (71.4%)	12 (70.6%)	15 (68.2%)	5 (55.5%)	1 (50.0%)
I don’t know	2 (11.8%)	0 (0%)	6 (54.5%)	0 (0%)	5 (29.6%)	0 (0%)	3 (33.3%)	0 (0%)
6. Do I need to have my AMDs witnessed or notarized?								
Yes	4 (23.6%)	5 (62.5%)	6 (54.5%)	4 (28.5%)	5 (29.4%)	12 (54.5%)	2 (22.3)	0 (0%)
No	10 (58.8%)	1 (12.5%)	4 (36.3%)	2 (14.3%)	5 (29.4%)	8 (36.4%)	3 (33.3%)	1 (50.0%)
I don’t know	3 (17.6%)	2 (25.0%)	1 (9.2%)	8 (57.2%)	7 (41.2%)	2 (9.1%)	4 (44.4%)	1 (50.0%)
7. Can a healthcare institution refuse to follow my AMDs?								
Yes	0 (0%)	0 (0%)	2 (18.1%)	0 (0%)	0 (0%)	0 (0%)	1 (11.2%)	1 (50.0%)
No	13 (76.4%)	5 (62.5%)	3 (27.4%)	9 (64.3%)	12 (70.6%)	15 (68.2%)	2 (22.2%)	1 (50.0%)
I don’t know	4 (23.6%)	3 (37.5%)	6 (54.5%)	5 (35.7%)	5 (29.4%)	7 (31.8%)	6 (66.6%)	0 (0%)
8. Do you know that the Supreme Court of India has given guidelines for AMDs								
Yes	4 (23.6%)	2 (25.0%)	6 (54.5%)	2 (14.3%)	5 (29.4%)	5 (22.7%)	3 (33.3%)	1 (50.0%)
No	10 (58.8%)	3 (37.5%)	1 (9.2%)	4 (28.5%)	5 (29.4%)	10 (45.5%)	2 (22.3%)	1 (50.0%)
I don’t know	3 (17.6%)	3 (37.5%)	4 (36.3%)	8 (57.2%)	7 (41.2%)	7 (31.8%)	4 (44.4%)	0 (0%)
9. At any time in your life, your health may deteriorate, and you may not be able to make decisions about your medical care. Have you ever considered the possibility that such a situation could arise?								
Yes	11 (64.7%)	2 (25.0%)	7 (63.7%)	5 (35.7%)	9 (53.0%)	13 (59.0%)	4 (44.5%)	1 (50.0%)
No	6 (35.3%)	6 (75.0%)	4 (36.3%)	9 (64.3%)	8 (47.0%)	9 (41.0%)	5 (55.5%)	1 (50.0%)
10. Will you consider "Do not resuscitate" (DNR) as one of the AMDS								
Yes	10 (58.8%)	6 (75.0%)	4 (36.3%)	6 (43.0%)	5 (29.4%)	16 (72.8%)	3 (33.3%)	2 (100%)
No	3 (17.6%)	2 (25.0%)	3 (27.4%)	2 (14.0%)	4 (23.6%)	6 (27.2%)	0 (0%)	0 (0%)
I don’t know	4 (23.6%)	0 (0%)	4 (36.3%)	6 (43.0%)	8 (47.0%)	0 (0%)	6 (66.7%)	0 (0%)
11. Whom would you like to choose as a healthcare proxy?								
Parents	4 (23.6%)	3 (37.5%)	6 (54.4%)	1 (7.1%)	5 (29.4%)	7 (31.8%)	2 (33.4%)	0 (0%)
Son/daughter	4 (23.6%)	5 (62.5%)	1 (9.2%)	6 (43.0%)	2 (11.7%)	9 (41.0%)	3 (22.2%)	2 (100%)
Close relative	5 (29.2%)	0 (0%)	3 (27.4%)	1 (7.1%)	0 (0%)	6 (27.2%)	3 (33.2%)	0 (0%)
Doctor	4 (23.6%)	0 (0%)	0 (0%)	1 (7.1%)	5 (29.4%)	0 (0%)	0 (0%)	0 (0%)
No healthcare proxy	0 (0%)	0 (0%)	1 (9.2%)	5 (35.7%)	5 (29.4%)	0 (0%)	1 (11.2%)	0 (0%)
12. If you had irreversibly lost your mental functions and were able to live only with artificial nutrition and breathing apparatus, would you like to continue life-sustaining treatment?								
Yes	6 (35.3%)	8 (100%)	5 (45.5%)	10 (71.4%)	8 (47.0%)	17 (77.3%)	2 (22.2%)	2 (100%)
No, let nature take its course	11 (64.7%)	0 (0%)	6 (54.5%)	4 (28.6%)	9 (53.0%)	5 (22.7%)	7 (77.8%)	0 (0%)
13. Do you want your decision or your family’s decision about AMDs to be considered concerning organ donation?								
Yes	13 (76.6%)	8 (100%)	4 (37.5%)	4 (28.5%)	12 (70.6%)	12 (54.5%)	3 (33.3%))	2 (100%)
No	0 (0%)	0 (0%)	3 (25.0%)	4 (28.5%)	0 (0%)	7 (31.8%)	0 (0%)	0 (0%)
I don’t know	4 (23.4%)	0 (0%)	4 (37.5%)	6 (43.0%)	5 (29.6%)	3 (13.6%)	6 (66.7%)	0 (0%)
14. Reason for not getting AMDs framed by you/your patient								
Ignorance	6 (35.3%)	5 (62.5%)	0 (0%)	1 (7.2%)	5 (29.4%)	5 (22.7%)	1 (11.2%)	1 (50.0%)
Costly	3 (17.6%)	0 (0%)	0 (0%)	0 (0%)	1 (5.9%)	2 (9.1%)	0 (0%)	0 (0%)
Involve legal proceeding	3 (17.3%)	1 (12.5%)	3 (27.4%)	5 (35.7%)	4 (23.6%)	4 (18.2%)	3 (33.3%)	1 (50.0%)
Never thought of framing AMDs	5 (29.4%)	2 (2.05%)	8 (72.6%)	8 (57.1%)	7 (41.2%)	11 (50.0%)	5 (55.5%)	0 (0%)
15. Will you help or motivate others, including family members, in framing their AMDs								
Yes	9 (52.8%)	5 (62.5%)	3 (27.4%)	3 (21.4%)	12 (70.6%)	5 (22.7%)	2 (22.2%)	1 (50.0%)
No	4 (23.6%)	0 (0%)	8 (72.6%)	6 (42.9.0%)	0 (0%)	14 (63.6%)	3 (33.4%)	1 (50.0%)
I don’t know	4 (23.6%)	3 (37.5%)	0 (0%)	5 (35.7%)	5 (29.4%)	3 (13.6%)	4 (44.4%)	0 (0%)

This study also found that no female or graduate participants were aware of the Supreme Court of India's guidelines on AMDs. Female participants had never anticipated a situation in which their health might deteriorate to the point where they could not make medical decisions, nor had they considered "Do not resuscitate" as an AMD option. All graduate participants (2, 100%), followed by government employees (5, 62.5%) and Hindus (7, 53.8%), selected their son or daughter as their healthcare proxy.

When all participants were questioned about their preferences in the event of irreversible loss of mental function and being managed by artificial means, all eight (100%) government employees and the majority of Hindus (10, 76.9%) preferred to continue life-sustaining treatment. Regarding organ donation, all participants (5, 100%) above 60 years, graduates (2, 100%), and government employees (8, 100%) strongly believed that their own or their family’s decision should be considered.

Among the reasons for not preparing AMDs for themselves or their patients, the majority of females, those educated up to high school, the unemployed, and participants aged 16-40 years had never considered it. In terms of willingness to help or encourage others, including family members, to prepare AMDs, participants above 60 years (5, 100%), followed by illiterate individuals (12, 70.6%) and government employees (5, 62.5%), showed the highest motivation (Tables 3-4).

## Discussion

To understand the perspectives of patients and their relatives regarding the formulation of AMDs and to evaluate any challenges encountered in this process, Dhru et al. conducted a study involving a highly educated, elite group of individuals [18]. Their findings showed that less than 30% were aware of AMDs. Medical professionals fared slightly better, with 63% demonstrating awareness. The study revealed that only 27.9% of respondents claimed to know AMDs, while 72.1% were unaware. Among those who were aware, 100 individuals had considered creating an AMD, but only 21 (18.2%) had done so. Overall, just 5.1% of respondents had completed an AMD. The majority (133, 58.8%) preferred their spouse as their healthcare proxy, 71 (30%) selected their parents, and 18 (8.0%) chose their family or children. Only three participants preferred a physician to act as their proxy if they could not make decisions [18].

Our study did not include healthcare professionals. However, among the participants in our research, the majority, particularly government employees (5, 62.5%) and Hindu community members (7, 53.8%), nominated their children as their healthcare proxy. This was followed by preferences for parents, close relatives, and doctors.

A population survey in Pune revealed that over 99% of respondents could indicate their preferred place of death, with 83% choosing to die at home [3]. Similarly, a Lebanese study involving middle-aged to older adults in primary care found that approximately 90% preferred truth-telling and healthcare autonomy. In comparison, 77% wished to document their healthcare values and preferences [19].

Our study questionnaire did not assess these parameters. In contrast, the majority of our participants were between 41 and 60 years old, were predominantly male, and had varying education levels: 17 (34%) illiterate, 22 (44%) literate, nine (18%) high school, and two (4%) graduates. Only three (6%) participants had a living will, while 10 (20%) participants intended to prepare one in the near future. A study by van Wijmen et al. found that Christianity-oriented groups were less likely than less religious groups to decline resuscitation near the end of life [20]. Similarly, Deepthi found that Asian Indian Hindus were more inclined to make independent decisions and reject life-sustaining measures [21]. Asian Hindus, or Indians in general, are more likely than non-Hispanic White individuals to refuse life-sustaining treatments and make independent decisions regarding AMDs. However, in our study, only two communities, 13 (26%) Hindu and 37 (74%) Sikh participants, were present, and we found that 20 (40%) participants did not want life extension. Among the Sikh community, 17 (45.9%) rejected life extension when they were unable to decide, compared to three (23.1%) in the Hindu community. The primary challenge lies in the fact that Indian conceptions of autonomy are relational rather than individualistic. In Indian filial culture, dependence on the family unit is both an expectation and a responsibility, with caregiving as a fundamental duty [22].

Limitations

As it was a critical care unit setting, representation from the community regarding their knowledge, perception, and attitude toward AMDs was absent. A more broadly based sample population should have been included to paint a true picture regarding AMDs. As the Supreme Court's judgment regarding AMDs is recent, there is the likelihood that the common people will still not be aware of the newly passed law regarding AMDs.

## Conclusions

The study highlights the low awareness and adoption of AMDs in India, even among highly educated individuals. While medical professionals demonstrated slightly higher awareness, only a small fraction of the general population had created an AMD. The findings suggest that cultural and familial influences play a significant role in decision-making, with most participants preferring family members, particularly spouses and children, as healthcare proxies rather than physicians.

Religious and cultural factors also shape perspectives on end-of-life care, with Hindu and Sikh participants exhibiting varying attitudes toward life-sustaining treatments. The study suggests that Indian society views autonomy through a relational lens rather than an individualistic one, where family involvement is central to medical decisions. This underscores the primary challenge in implementing AMDs, balancing individual preferences with deeply rooted familial and cultural values. Additionally, comparisons with global studies indicate that while preferences for autonomy and end-of-life planning exist in other regions, Indian perspectives are influenced by the country’s strong familial bonds and caregiving responsibilities. Despite these challenges, most participants recognized the benefits of AMDs for Indian society, signaling potential for greater acceptance with increased awareness and education.
